# The Assessment of Immune Fitness

**DOI:** 10.3390/jcm12010022

**Published:** 2022-12-20

**Authors:** Joris C. Verster, Aletta D. Kraneveld, Johan Garssen

**Affiliations:** 1 Division of Pharmacology, Utrecht Institute for Pharmaceutical Sciences, Utrecht University, 3584CG Utrecht, The Netherlands; 2 Centre for Human Psychopharmacology, Swinburne University, Melbourne, VIC 3122, Australia; 3 Global Centre of Excellence Immunology, Nutricia Danone Research, 3584CT Utrecht, The Netherlands

**Keywords:** immune fitness, immune functioning, assessment, single-item scale, ISQ, biomarkers

## Abstract

Immune fitness (i.e., adequate functioning of the immune system) is essential to maintain health, prevent and resolve disease, and improve quality of life. This article provides an overview of how to assess immune fitness. It discusses how a single-item rating scale can be used to assess immune fitness. The scale can be used in conjunction with a single “yes” or “no” question asking whether the individual is experiencing reduced immune fitness. Retrospective assessments can be complemented with the Immune Status Questionnaire (ISQ) to provide more insight into the type and frequency of experiencing specific immune-related complaints. Momentary assessments of immune fitness can be complemented with biomarker measurements in body fluids. As individuals may be unaware of systemic inflammation (e.g., biomarker concentrations outside the normal range), it remains critical to combine immune fitness assessments with biomarker measurements of immune functioning.

## 1. Introduction

Immune fitness refers to the body’s capacity to respond to health challenges (such as infections) by activating an appropriate immune response, which is essential to maintain health, prevent and resolve disease, and improve quality of life. Immune fitness (i.e., adequate functioning of the immune system) is essential to reduce the likelihood of developing noncommunicable diseases (NCDs), such as cardiovascular diseases, cancers, respiratory diseases, and diabetes [1,2]. NCDs are responsible for 71% of all deaths worldwide [3]. In addition, the immune system is involved in communicable diseases, including viral infections, such as the 2019 coronavirus disease (COVID-19) [4]. Reduced immune fitness has a significant negative impact on mood, perception of health, daily activities, interactions with others, and quality of life [5,6].

Immune fitness refers to a person’s perception of the extent to which he/she is capable of preventing and resolving disease through adequate immune functioning. This perception is vital because it can determine whether an individual decides to seek medical advice or undertake actions to adjust lifestyle factors, such as adopting a healthier diet or increasing physical activity. Thus, experiencing reduced immune fitness is an important signal for an individual to take action. Objective assessments of immune functioning that determine whether biomarkers (e.g., cortisol) or physiological parameters (e.g., blood pressure) are outside the normal (healthy) range are very informative in this regard. However, these assessments are usually conducted only after seeking medical attention. The individual may not notice changes in biomarkers (e.g., cholesterol) or physiological parameters (e.g., hypertension). Due to this lack of awareness, action is often not taken until a physician discovers these changes. For several other conditions, there are no adequate biomarkers available (e.g., depression and poor sleep quality). Nevertheless, these related signs and symptoms often contribute to an individual’s judgment of his/her immune fitness and decision to visit a physician. It is important to assess immune fitness in both research studies and clinical practice. This article discusses how to measure immune fitness.

**Figure 1:**
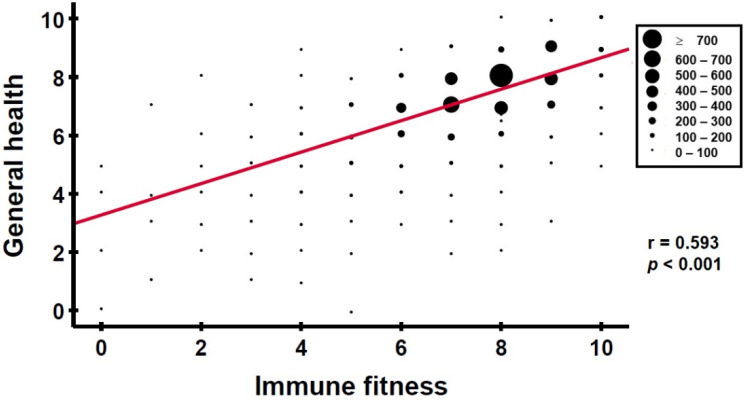
Relationship between general health and immune fitness. General health and immune fitness were both scored on a single-item rating scale ranging from 0 (very poor) to 10 (excellent). Data for *n* = 4272 individuals, taken from references [7,8,9,10,11,12].

Since adequate immune fitness is a prerequisite for maintaining health and preventing disease, assessments of the two concepts of health and immune fitness are significantly related. Figure 1 shows this relationship for 4272 individuals [7,8,9,10,11,12].

## 2. Assessment of Immune Fitness

**Figure 2:**
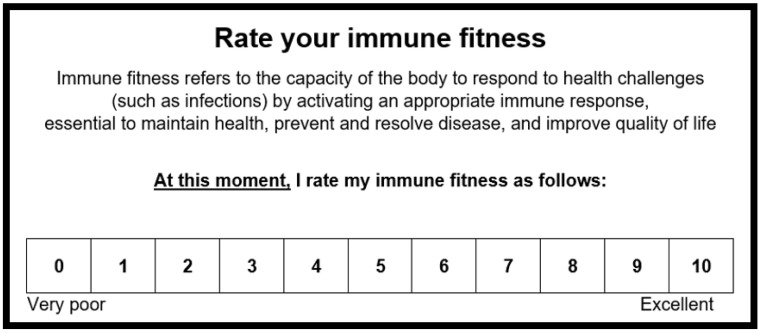
Assessment of immune fitness.

Immune fitness can be assessed with a single-item patient-reported outcome (PRO) measure (see Figure 2). The 11-point scale ranges from 0 (very poor) to 10 (excellent). Depending on the individual’s knowledge/education level, a short description defining “immune fitness” can be included.

This single-item assessment of immune fitness was first used in 2015 in survey research conducted at Utrecht University in the Netherlands [7,8,9]. Thereafter, the measure has been successfully used in a series of studies. These studies revealed that the level of immune fitness differentiated individuals with a variety of health conditions, such as individuals with and without NCDs (e.g., cardiovascular or pulmonary diseases, diabetes) [13], or individuals with and without self-reported impaired wound healing [14]. Furthermore, immune fitness has been shown to be impacted differently by psychosocial characteristics, such as whether an individual lived alone or with other people [15]. Another study demonstrated that going on a holiday positively affected immune fitness [16]. Several studies have revealed that immune fitness is significantly correlated with many health outcomes, including irritable bowel syndrome (IBS) symptoms [17], insomnia/sleep quality [18], and number and severity of COVID-19 symptoms [4]. Significant correlations have also been shown between immune fitness and mood (e.g., anxiety, depression, and stress) [19], as well as quality of life [10]. Finally, immune fitness correlated significantly with certain health risk factors, such as body mass index (BMI) [20] and weekly alcohol consumption [19], and protective factors, such as amount of vigorous physical activity per week [21], mental resilience [10], optimism, and ability to cope with stress [19]. The outcomes of these studies demonstrate that immune fitness plays a crucial role in an individual’s wellbeing and quality of life, both in health and disease.

**Figure 3:**
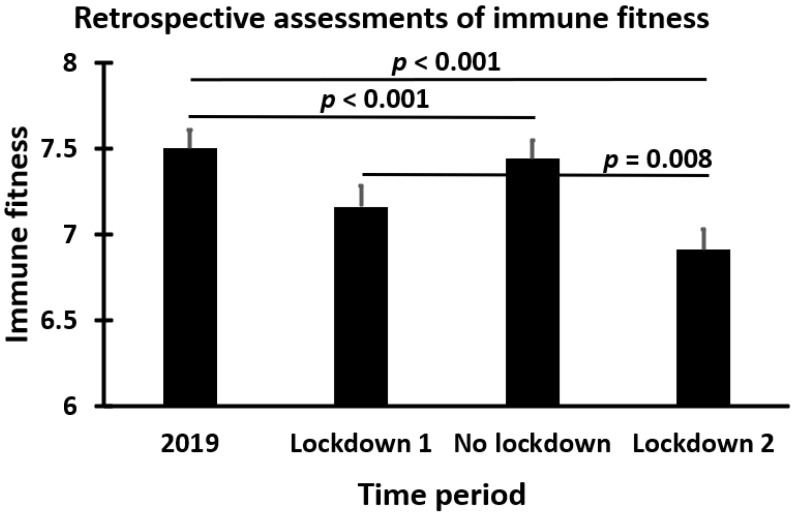
Retrospective assessments of immune fitness. The immune fitness of *n* = 254 Utrecht University students was assessed with a single-item scale ranging from 0 (very poor) to 10 (excellent). The assessed time periods were 15 March 2019–11 May 2020 (Lockdown 1), 12 May 2020–31 October 2020 (No lockdown), and 1 November 2020–1 April 2021 (Lockdown 2). Means and standard errors (SE) are shown. Differences between the time periods were considered statistically significant if *p* < 0.0083 (applying Bonferroni’s correction for multiple comparisons). Data were taken from Hendriksen et al. [5,22].

In addition to momentary and real-time assessments of immune fitness, assessments can also be made retrospectively. Retrospective assessments are time-locked assessments whose outcomes may differ from occasion to occasion. For example, in a study of Utrecht University students, participants rated their immune fitness for various time periods during the COVID-19 pandemic [5,22]. The outcomes are summarized in Figure 3. The differences in immune fitness ratings underline the importance of setting a clear time period when assessing immune fitness retrospectively. Thus, the single-item immune fitness assessment can be a quick tool for making assessments for various periods in time.

## 3. Reduced Immune Fitness

**Figure 4:**
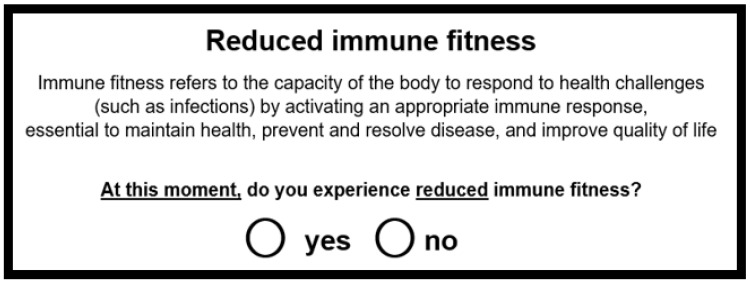
Assessment of reduced immune fitness.

The question “At this moment, do you experience reduced immune fitness?”, with the answering options “yes” or “no”, is a direct and very effective way to determine whether a subject is experiencing reduced immune fitness (see Figure 4).

The question to assess whether an individual was experiencing reduced immune fitness was first used in 2015 by Donners et al. [6]. In a sample of 574 students, the researchers found that students who reported reduced immune fitness also reported significantly higher levels of insomnia, sleep apnea, and circadian rhythm disorder complaints. In addition, individuals with reduced immune fitness reported significantly poorer daily functioning and a significantly lower quality of life compared to individuals with normal immune fitness. In most subsequent studies, the question on reduced immune fitness was incorporated together with the single-item immune fitness assessment.

**Figure 5:**
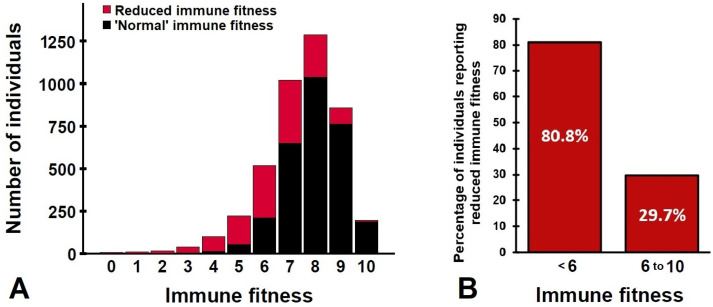
Reduced immune fitness. Shown are the distribution (**A**) and percentages (**B**) of individuals reporting normal or reduced immune fitness. Data for *n* = 4272 individuals, taken from references [7,8,9,10,11,12].

Reduced immune fitness is a subjective self-assessment in which subjects compare their current immune fitness level with the level of immune fitness they consider themselves as “normal”. It is important to note that researchers or physicians cannot make a judgment on reduced or normal immune fitness by simply considering the momentary immune fitness rating of an individual. It would be incorrect to label all cases with immune fitness ratings below 6 as individuals with reduced immune fitness. For example, an individual with a “normal” immune fitness rating of 9 who reports a momentary immune fitness rating of 7 may also report reduced immune fitness, even though the absolute rating is still above 6. Figure 5 shows that a considerable number of individuals (in this sample, approximately 29.7%) reported reduced immune fitness, while their momentary immune fitness rating was 6 or higher. On the other hand, there is also a subsample of individuals who have a normal immune fitness score below 6 and therefore do not report reduced immune fitness (19.2% of the current sample). In other words, one cannot conclude reduced immune fitness based on absolute immune fitness ratings; but must directly ask the individual whether or not he experiences reduced immune fitness.

## 4. Multiple-Item Scales to Assess Immune Functioning: The IFQ and ISQ

Two multiple-item questionnaires are available to retrospectively assess immune fitness. Reed et al. [23] developed the Immune Function Questionnaire (IFQ). This questionnaire comprises 19 signs of weakened immune system functioning. The items include sore throat, headaches, flu, runny nose, coughing, cold sores, boils, mild fever, warts/verrucas, pneumonia, bronchitis, sinusitis, sudden high fever, ear infection, diarrhea, meningitis, eye infection, sepsis, and long-healing injuries. Individuals indicate whether they have experienced one or more of these signs using a 5-point Likert-type scale, with the answering possibilities “Never”, “Once or twice”, “Occasionally”, “Regularly”, and “Frequently”, with scores ranging from 0 to 4. The total score ranges from 0 to 76; the higher the score, the worse the immune function. Thus, the IFQ assesses the type and frequency of immune-related complaints but does not take into account the duration, severity, and impact of the complaints or the potential for coping. Reed et al. [23] reported that the total IFQ score correlated significantly (r = 0.345, *p* < 0.001) with the outcome of the General Health Questionnaire (GHQ-28) [24]. Although modest (in the range from r = 0.2 to r = 0.25), they reported significant correlations between IFQ score and depression, anxiety, and sleep quality [23].

To our knowledge, the IFQ has not been used in clinical practice, and its use for research purposes has been limited to two studies by our group. These studies found significant correlations between IFQ scores and mental resilience, general health, quality of life [10], and autism spectrum quotient (AQ) ratings [25]. Some shortcomings of the IFQ were noted when conducting these studies. Specifically, the IFQ does not include some other common immune-related complaints, such as muscle and joint pain or skin problems (e.g., acne and eczema), whereas it does include relatively uncommon items, such as meningitis. Given this, Wilod Versprille et al. [26] developed the Immune Status Questionnaire (ISQ). The IFQ items were reconsidered, and other items were added. After regression analysis, the original list of 23 immune-related complaints was reduced to seven items [26]. The ISQ comprises seven items, including “common cold”, “diarrhea”, “sudden high fever”, “headache”, “muscle and joint pain”, “skin problems (e.g., acne and eczema)”, and “coughing”. Individuals can indicate how frequently they experienced these immune-related complaints during the past year. The answering options are “never”, “sometimes”, “regularly”, “often”, and “(almost) always”. The sum score of the ISQ is then recoded into a 0 (very poor) to 10 (excellent) scale. This can be done manually, but automated computer scripts have also been published for this purpose [27].

The ISQ is used worldwide in both research and clinical practice and has been translated into various languages, including Arabic [28], Dutch [26], English [29], German [30], Italian [31], and Indonesian [32]. Similar to the single-item assessment of immune fitness, ISQ scores have been shown to be significantly associated with health correlates, such as IBS [18], depression, anxiety, stress [28,31], risky decision making [31], presence and severity of COVID-19 symptoms [4], dietary changes [33,34], attaining a healthy diet [35], and BMI [20].

## 5. Comparison of the Single-Item Assessment of Immune Fitness and the ISQ

The single-item scale allows an individual to self-assess immune fitness. A short description of immune fitness is provided to aid in this assessment. When rating immune fitness, it is hypothesized that the individual takes into account (1) the type and number of possible immune-related complaints being experienced, (2) the frequency of experiencing these symptoms, (3) the severity of these complaints, (4) the duration of the complaints until they are resolved, (5) how these complaints are impacting their daily activities and interactions with others (e.g., driving a car, work performance, visiting family and friends), and (6) the individual’s ability to counteract or cope with these complaints (e.g., mental resilience, personality). Then, taken together, these components determine the reported immune fitness score. With the single-item scale, a global assessment of immune fitness can be made, regardless of the type and nature of immune-related complaints. Thus, the single-item approach provides a global assessment that evaluates the entire constellation of immune fitness, regardless of the individual components contributing to it, in terms of the presence of immune-related complaints, their severity, and their impact. As such, the single-item assessment of immune fitness satisfies the criteria set in the FDA guidelines for the development of an effective patient-reported outcome measure [36]. A single-item scale can provide a real-time or retrospective, burden-free, directly available outcome that can be used in survey research, randomized clinical trials, and clinical practice [37].

The single-item immune fitness assessment differs in many ways from the multiple-item assessments of the IFQ and ISQ. First, with the IFQ and ISQ, only retrospective assessments can be made for a set period of time. Momentary, real-time assessments cannot be conducted. The original time period for assessments with the ISQ was set at one year. This period of time was chosen to allow sufficient time for immune-related complaints to occur. However, this time period can be adjusted depending on the requirements of a particular study. For example, one study adjusted the time period of assessment to cover an 18-month COVID-19 pandemic period in Germany [30], whereas another study shortened the time period to 6 months to cover a lockdown-free period in the Netherlands [35]. Second, the IFQ and ISQ comprise items assessing the frequency of the occurrence of a limited number of selected immune-related complaints. Per definition, by including a selection of items in a questionnaire, other items are omitted and not considered. Thus, the IFQ and ISQ do not cover all possible immune-related complaints. Third, the IFQ and ISQ do not assess the duration and severity of the selected immune-related complaints, nor do they assess the impact of experiencing the complaints or consider possibilities for coping with or counteracting them.

**Figure 6:**
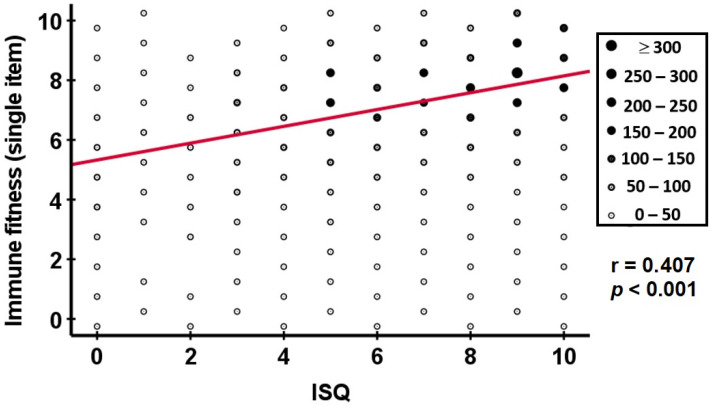
Correlation between the single-item immune fitness scale and the ISQ. Spearman’s correlation is depicted (red line). Dot sizes are proportional to the number of individuals. Abbreviation: ISQ = Immune Status Questionnaire. Data from *n* = 3748 individuals, taken from references [10,11,12,13,16,33,34,38].

A summary of the commonalities and differences between the assessments is presented in Table 1. While it is assumed that an individual incorporates all six aspects of immune-related complaints in his or her evaluation when rating immune fitness with the single-item scale or the question on reduced immune fitness, the IFQ and ISQ inquire only about the frequency of the occurrence of a selected number of immune-related complaints and do not consider severity, duration, impact, and coping ability. It is therefore understandable that assessments of immune fitness based on a single-item scale versus multiple-item scales have different outcomes and are thus not interchangeable. Indeed, Figure 6 shows only a modest correlation between the single-item assessment of immune fitness and the ISQ in 3748 individuals (r = 0.407, *p* < 0.001) [10,11,12,13,16,33,34,38]. However, the positive correlation does show whether immune-related complaints are experienced frequently, which is commonly reflected in a lower single-item immune fitness score. A previous study found a correlation of comparable magnitude (r = −0.423) between the IFQ and the single-item assessment of immune fitness [10].

## 6. Biomarkers of Immune Fitness?

It would be ideal if a biomarker or set of biomarkers were available to objectively measure immune fitness. Biomarkers or objective assessments are available for some health outcomes (e.g., disease state, mental or physical condition). For example, weight and height, body temperature, blood pressure, and heart rate can be objectively determined. An example of an effective biomarker is blood glucose measurement to aid in the self-treatment of diabetes (e.g., to adjust insulin dosing). However, for most health outcomes in clinical medicine and psychiatry, there are no objective biomarkers. For example, anxiety, depression, stress, and sleep quality can only be assessed using a PRO. The pathophysiological background of these conditions is complex and not fully understood. Consequently, there is no single biomarker or set of biomarkers available that accurately reflects overall health outcomes. However, some biomarkers are clearly related to health outcomes and can therefore be considered proxy measures. For example, a study found that 57 of 150 biomarkers that were assessed in blood or urine significantly correlated with self-reported general health [39]. However, the strength of the significant correlations, in the range from r = 0.2 to r = 0.4, was only modest [39]. This is understandable, as biomarkers are usually considered in isolation from the complex system to which they belong.

Furthermore, in the case of immune fitness, research has revealed that it is not possible for a single immune system biomarker (e.g., a marker of systemic inflammation such as C-reactive protein (CRP) or a cytokine) to adequately reflect immune fitness. The immune system is simply too complex and dynamic for a single biomarker or a small set of biomarkers to represent its overall function. Over 150 cytokines (i.e., small signaling molecules and other immune cells) [39] work together to orchestrate immune responses [40]. Assessing a single cytokine by itself is unlikely to accurately reflect the activity of the immune system as a whole. Therefore, correlations between biomarker concentrations and immune fitness assessments are expected to be modest at best. Only one study has directly compared the two. Petrie et al. [41] examined 20 healthy volunteers and found that global ratings of their immune functioning did not significantly correlate with serum immunoglobulin A (IgA), IgG, and IgM antibodies or with the cluster of differentiation 3 (CD3), CD4, CD8, and CD16 lymphocytes. Instead, feelings of vigor and fatigue were the main determinants of individuals’ perceptions of their immune functioning.

It is important to stress that biomarkers of systemic inflammation are a proxy for immune fitness. This means that while biomarkers may assess processes related to the immune system, they do not measure the overall concept of immune fitness. Therefore, individuals may experience reduced immune fitness, even though they have no objective (biomarker) signs of systemic inflammation. Alternatively, individuals may show objective signs of systemic inflammation but at the same time report adequate immune fitness. The latter was illustrated in a recent study by van Oostrom et al. [35], who assessed both C-reactive protein (CRP) in saliva and momentary immune fitness. They further asked participants whether (1) they experienced reduced immune fitness and (2) they had any inflammatory conditions. Of the 103 participants, 21 reported having inflammatory conditions. The salivary CRP levels of those reporting inflammatory conditions were significantly higher than those of the 82 participants who reported no inflammatory conditions (mean ± SD: 251.2 ± 281.1 versus 132.1 ± 161.2, respectively, *p* = 0.018). However, only five individuals in the sample reported reduced immune fitness. The most important reason for this discrepancy is that individuals without objective signs of systemic inflammation may still report reduced immune fitness (for example, when a “normal” immune fitness score of 9 is reduced to 7) and vice versa (individuals with objective systemic inflammation who report adequate immune fitness). Further analysis revealed no significant correlations (r < 0.2) between the single-item assessment of immune fitness and salivary concentrations of CRP, IL-1β, and IL-8 for the sample as a whole [35]. Together, these findings support the notion that although biomarkers may measure clinically relevant aspects of immune functioning (i.e., values outside the normal range), they do not adequately represent the overall concept of immune fitness. Nevertheless, biomarker assessments remain of critical importance to objectively assess the possible presence of systemic inflammation. If one is interested in the patient’s experience, immune fitness should be assessed with the single-item rating or the ISQ. Systemic inflammation can, but does not necessarily is, a consequence of reduced immune fitness. If one is particularly interested in systemic inflammation (i.e., whether the concentration of the biomarker is outside the normal range), biomarker assessment would be relevant.

## 7. Discussion

**Figure 7:**
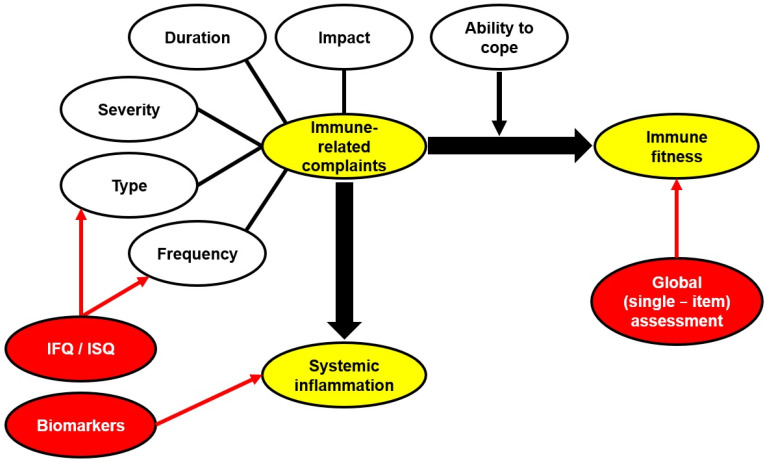
Assessment of immune fitness. Characteristics of immune-related complaints (e.g., severity and duration) are shown in white ovals. Depending on the ability to cope, these characteristics determine immune fitness (indicated by a black arrow). In addition, immune-related complaints can be associated with systemic inflammation (indicated by a black arrow). The red arrows indicate which domains are assessed by the IFQ/ISQ (type and frequency of immune-related complaints), biomarkers (systemic inflammation), and the global (based on a single item) assessment (immune fitness). Abbreviations: IFQ = Immune Function Questionnaire, ISQ = Immune Status Questionnaire.

Several aspects should be considered in the assessment of immune fitness, including (1) the type and number of possible immune-related complaints being experienced, (2) the frequency of experiencing these symptoms, (3) the severity of these complaints, (4) the duration of the complaints until they are resolved, (5) how these complaints impact daily activities and interactions with others, and (6) the individual’s ability to counteract or cope with these complaints. Figure 7 illustrates these aspects of immune fitness. From this review, it appears that the single-item immune fitness scale is most suitable for assessing immune fitness and that reduced immune fitness can be determined with a single question. Multiple-item scales, such as the ISQ for retrospective assessment over specific time periods, as well as biomarker measurements of systemic inflammation for momentary assessments, can complement and support the assessment of immune fitness and provide more insight into which immune-related processes are affected.

The assessment of momentary and retrospective immune fitness can be conducted using a single-item PRO. This 0 (very poor) to 10 (excellent) rating scale of immune fitness is a valid, reliable, and cost- and time-effective measure. The single-item approach provides a global assessment that evaluates the entire constellation of immune fitness, regardless of the individual components contributing to it, in terms of the presence of immune-related complaints, their severity, and their impact. For the same reasons, reduced immune fitness can most accurately be assessed with a single question with the options “yes” or “no”. The single-item immune fitness assessment takes into account the type, presence, number, frequency, severity, duration, impact, and coping ability for immune-related complaints. However, the global rating provides no information on these characteristics.

The assessment of biomarkers serves an important purpose. Biomarkers are valuable tools for objectively determining whether immune functioning is deviating from normal conditions, which can be established when biomarker concentrations fall outside the normal concentration range expected for healthy individuals. Based on these assessments, a physician can make a diagnosis and select a treatment for the patient. Individuals may be unaware of changes in biomarkers of the immune system, similar to individuals not usually being aware of high cholesterol levels or hypertension until objective assessments are made. Thus, the individual may report adequate immune fitness while objectively having systemic inflammation. In such instances, the assessment of biomarkers remains of critical importance.

For future research, it would be interesting to develop a more detailed immune fitness questionnaire that also assesses the severity, duration, impact, and ability to cope with immune-related complaints. Including all six characteristics shown in Table 1 would give a more complete background overview, making the global assessment easier to interpret. It would also be important to include items on the impact of immune-related complaints and methods of coping with these complaints. The latter is important, as they may differ between immune-related complaints. In other words, the various immune-related complaints may differ in the amount of impact they have on an individual’s daily activities. For example, work performance is likely to suffer more from a headache than from a common cold. Also, perceptions of health and interpretations of the concepts of health and disease may be different for specific subgroups, such as between young and old individuals [42,43] or between men and women [44,45]. Therefore, the impact of immune-related factors and the role of coping strategies should not be underestimated and should be included in a newly developed questionnaire.

In clinical practice, immune fitness assessments could begin with the single-item assessment. When scores are low or individuals report reduced immune fitness, then they could move on to complete a new, more elaborate questionnaire to provide more insight into the nature of their immune fitness rating. Such a new questionnaire would also be helpful for physicians in determining which biomarker or physiological assessments would be useful to conduct. Combining these forms of assessment would be a time- and cost-effective way of comprehensively evaluating immune fitness.

Finally, the use of the adjectives “perceived” and “subjective” for PROs of immune fitness gives the false impression that there are also objective measures for these conditions. Therefore, it is not common practice to refer to perceived or subjective anxiety or perceived or subjective depression. The adjectives are omitted, and one simply refers to anxiety or depression. Similarly, there are no objective measures of immune fitness. Therefore, it is also proposed to abandon the use of these adjectives for PROs of immune fitness and simply refer to ‘immune fitness’ in future publications.

## 8. Conclusions

A single-item rating scale can be used to assess immune fitness. The scale can be used in conjunction with a single question that asks individuals whether they are experiencing reduced immune fitness. Retrospective assessments can be complemented with the Immune Status Questionnaire (ISQ) to provide more insight into the type and frequency of specific immune-related complaints an individual is experiencing. Momentary assessments of immune fitness can be complemented with biomarker assessments. As individuals may be unaware of objective changes in immune functioning (i.e., biomarker concentrations outside the normal range), it remains critical to combine assessments of immune fitness with biomarker measurements of immune functioning.

## Figures and Tables

**Table 1:** Comparison of assessment methods.

Different Aspects of Immune-Related Complaints		Immune Fitness (Single-Item)	Reduced Immune Fitness (Yes/No)	IFQ/ISQ
1	Type and number	√	√	√
2	Frequency	√	√	√
3	Severity	√	√	X
4	Duration	√	√	X
5	Impact	√	√	X
6	Ability to cope	√	√	X

√ = incorporated in the measure; X = not incorporated in the measure. Abbreviations: IFQ = Immune Function Questionnaire, ISQ = Immune Status Questionnaire.

## Data Availability

Not applicable.
